# SRR intronic variation inhibits expression of its neighbouring SMG6 gene and protects against temporal lobe epilepsy

**DOI:** 10.1111/jcmm.13473

**Published:** 2018-01-24

**Authors:** Hua Tao, Xu Zhou, Qian Xie, Zhonghua Ma, Fuhai Sun, Lili Cui, Yujie Cai, Guoda Ma, Jiawu Fu, Zhou Liu, You Li, Haihong Zhou, Jianghao Zhao, Yanyan Chen, Hui Mai, Ying Chen, Jun Chen, Wei Qi, Chaowen Sun, Bin Zhao, Keshen Li

**Affiliations:** ^1^ Department of Neurology Affiliated Hospital of Guangdong Medical University Zhanjiang Guangdong China; ^2^ Guangdong Key Laboratory of Age‐related Cardiac and Cerebral Diseases Guangdong Medical University Zhanjiang Guangdong China; ^3^ Clinical Research Center Guangdong Medical University Zhanjiang Guangdong China; ^4^ Emergency Department Affiliated Hospital of Guangdong Medical University Zhanjiang Guangdong China; ^5^ Department of Neurology Beijing Tongren Hospital Capital Medical University Beijing China; ^6^ Department of Neurology the First People's Hospital of Pingdingshan Pingdingshan Hebei China; ^7^ Department of Neurology Central People's Hospital of Zhanjiang Zhanjiang Guangdong China; ^8^ Institute of Neurology Guangdong Medical University Zhanjiang Guangdong China; ^9^ Stroke Center Neurology& Neurosurgery Division Clinical Medicine Research Institute & the First Affiliated Hospital, Jinan University Guangzhou Guangdong China

**Keywords:** serine racemase, SMG6, N‐methyl‐D‐aspartate receptor, temporal lobe epilepsy

## Abstract

D‐serine is a predominant N‐methyl‐D‐aspartate receptor co‐agonist with glutamate, and excessive activation of the receptor plays a substantial role in epileptic seizures. Serine racemase (SRR) is responsible for transforming L‐serine to D‐serine. In this study, we aimed to investigate the genetic roles of SRR and a neighbouring gene, nonsense‐mediated mRNA decay factor (SMG6), in temporal lobe epilepsy (TLE). Here, a total of 496 TLE patients and 528 healthy individuals were successfully genotyped for three SRR tag single nucleotide polymorphisms. The frequencies of the GG genotype at rs4523957 T > G were reduced in the TLE cases in the initial cohort (cohort 1) and were confirmed in the independent cohort (cohort 2). An analysis of all TLE cases in cohort 1 + 2 revealed that the seizure frequency and drug‐resistant incidence were significantly decreased in carriers of the GG genotype at rs4523957. Intriguingly, the activity of the SMG6 promoter with the mutant allele at rs4523957 decreased by 22% in the dual‐luciferase assay, and up‐regulated expression of SMG6 was observed in an epilepsy rat model. This study provides the first demonstration that the GG genotype is a protective marker against TLE. In particular, variation at rs4523957 likely inhibits SMG6 transcription and plays a key role against susceptibility to and severity of TLE. The significance of SMG6 hyperfunction in epileptic seizures deserves to be investigated in future studies.

## Introduction

According to the World Health Organization, approximately 50 million people live with epilepsy worldwide, and nearly, one‐third of these individuals are severely affected by intractable seizures. Notably, an increasing amount of evidence indicates that temporal lobe epilepsy (TLE), the most common partial epilepsy, is a primary origin of intractable seizures, but progress in its clinical treatments has always lagged far behind expectations 1, 2, 3, 4. Because TLE is a complex disease, determination of its common mechanisms might be a promising shortcut for the identification of key targets for conquering TLE, particularly TLE‐related intractable seizures.

As is well known, abnormalities in various neurotransmitters and receptors and pathological remodelling of synaptic junctions have been implicated in the pathogeneses of TLE. Nevertheless, all epileptic seizures are ultimately triggered by an imbalance of excitatory and inhibitory signals in the brain, such as overactivation of excitatory processes and/or attenuation of inhibitory processes. Notably, the N‐methyl‐D‐aspartate (NMDA) ionotropic glutamate receptor can bind glutamate, a primary excitatory neurotransmitter, and activate rapid excitatory neurotransmission in a cascade of physiological activities, such as physical development and memory. However, excessive activation of the NMDA receptor usually plays a substantial role in epileptic seizures 5, 6, 7. Currently, direct blockade of the NMDA receptor has yielded anti‐epileptic effects 8, 9, 10, but this treatment is susceptible to undesirable side effects because of extensive interference in physiological activities 11. In recent years, molecules upstream and downstream of the NMDA receptor began to be studied as potential targets with the aim of fine‐tuning NMDA receptor‐mediated excitatory disorders.

D‐serine is transformed from L‐serine by SRR and acts as a predominant NMDA receptor co‐agonist with glutamate 12, 13, 14. At present, SRR inhibition is considered a promising approach for selectively reducing coactivation of the NMDA receptor and for use in conditions in which NMDA receptor‐mediated excitotoxicity plays a pathological role 11, 15. In fact, decreased susceptibility to epileptic seizures has been observed in SRR‐knockout mice 16. In an epilepsy animal model, SRR was found to be involved in reactive astrogliosis, a pathological hallmark of TLE 17. Together, these findings support the significance of SRR hyperfunction in TLE.

Single nucleotide polymorphisms (SNPs) are the most common component of genetic variations in humans and play a widespread role in fine‐tuning gene expression. Previous studies have demonstrated that rs408067 SNPs affect the transcriptional activity of the SRR gene promoter and susceptibility to schizophrenia characterized by dysregulation of the NMDA receptor 18. The available evidence raises the question of whether the variation at rs408067 G > C affects TLE by promoting NMDA receptor‐mediated excitatory processes. In contrast, an analysis of HapMap data for Chinese Hans using Haploview 4.2 identified three tag SNPs, namely, rs4523957, rs8081273 and rs7222251, as representative SNPs in the SRR gene. Among these three intronic SNPs, rs4523957 is also located in the promoter region of SMG6, a neighbouring gene of SRR that is responsible for the synthesis and maintenance of telomere repeats and nonsense‐mediated mRNA decay (NMD). Some telomere abnormalities and nonsense mutations have been reported to be associated with seizures 19, 20, 21, but direct evidence of the role of SMG6 in epileptic seizures is lacking. In this study, we aimed to identify the genetic association of SRR with TLE through an analysis of the above‐mentioned representative SNPs and supply preliminary evidence of the role of SMG6 in epileptic seizures.

## Materials and methods

### Human subject enrollment

All experiments on human subjects performed in this study were approved by the Ethics Committees of the Affiliated Hospital of Guangdong Medical University, the First Affiliated Hospital of Harbin Medical University, the Central People's Hospital of Zhanjiang, the First People's Hospital of Pingdingshan and Beijing Tongren Hospital, affiliated with Capital Medical University, and were conducted in accordance with the Declaration of Helsinki. Informed consent documents were signed at the time of subject enrollment.

A total of 335 TLE patients and 325 healthy individuals from the First Affiliated Hospital of Harbin Medical University, the Affiliated Hospital of Guangdong Medical University and Beijing Tongren Hospital, affiliated with Capital Medical University, were consecutively enrolled in cohort 1. To confirm the significant statistics observed in the initial cohort, a validating cohort (cohort 2) composed of 161 patients with TLE and 203 healthy individuals consecutively recruited from the First People's Hospital of Pingdingshan and the Central People's Hospital of Zhanjiang was also established. All human subjects in cohorts 1 and 2 are Han Chinese, and the combined cohort (cohort 1 + 2) included 496 patients with TLE and 528 healthy individuals.

The gender, age, age at onset, disease duration and severity of disease (seizure frequencies and drug response) of the participants were documented by field or telephone investigation after enrollment. The inclusion criteria for TLE were mainly based on typical temporal auras and temporal discharges at onset determined through video‐electroencephalograph (V‐EEG), and subjects who failed to be genotyped were excluded. According to the definition of drug‐resistant epilepsy proposed in 2010 by the Commission of the International League Against Epilepsy 22, the responses to drug treatments in the TLE cases were classified as follows: drug‐resistant patients were determined based on observations of the absence of a significant change or reduction in the seizure frequency (<60%) or even augmentation after 1‐year treatment with a schedule of at least two tolerated and properly selected anti‐epileptic drugs; the remaining individuals were considered drug‐sensitive patients.

### DNA preparation and genotyping

First, 2‐ml peripheral venous blood samples were collected from each human subject. DNA was extracted using a Genomic DNA Extraction Kit (Tiangen Biotech, Beijing, China) and genotyped for the four SRR SNPs (rs408067, rs4523957, rs8081273 and rs7222251) with an ABI PRISM SNaPshot system (Applied Biosystems, Carlsbad, CA, USA). The primers used in the multiplex polymerase chain reaction (PCR) for the amplification of the four target fragments (184, 330, 310 and 344 base pairs, respectively) were as follows: rs408067, 5′‐GCCCTCCCTTTCCGAACGAC‐3′ (forward primer) and 5′‐TCTGTCAACCCGAGTCCCAGAC‐3′ (reverse primer); rs4523957, 5′‐AGGAGACATGGAGAAGGGGTACAA‐3′ (forward primer) and 5′‐AAACCAGGAGGCAGGTTGCAGT‐3′ (reverse primer); rs8081273, 5′‐ATATTGTGGTGCCCCAGACAGC‐3′ (forward primer) and 5′‐GGGAGAGAAGTTGGAGTTGGTGTTG‐3′ (reverse primer); and rs7222251, 5′‐TCTTGATCTCCCTACCTCGTGATCC‐3′ (forward primer) and 5′‐AAAAGAAAATATGGGCCTGGTGTG‐3′ (reverse primer). In addition, the primers used for extension in SNaPshot PCR were 5′‐TTTTTTTTTTTTTTTTTTACCTCTGCGCACGCGCAGC‐3′ (rs408067), 5′‐TTTTTTTTTTTTTTTTTTTTTTTTCTACRTTGGTGGAAATTTTTGAATTATCA‐3′ (rs4523957), 5′‐TTTTTTTTTTTTCTCAGCCTCCTTTGTGGTTTCC‐3′ (rs8081273) and 5′‐ACTGCGCCCCACCCCATTT‐3′ (rs7222251).

The multiplex PCR reaction mix contained 1× HotStarTaq buffer, 3.0 mM Mg^2+^, 0.3 mM dNTP, 1 U of HotStarTaq polymerase, 1 μl of primer mix and 1 μl of the DNA template. Amplification PCR was performed as follows: 1 cycle of 95°C/2 min.; 11 cycles of 94°C/20 sec., 65°C/40 sec. and 72°C/90 sec.; 24 cycles of 94°C/20 sec., 59°C/30 sec. and 72°C/90 sec.; and 1 cycle of 72°C/2 min. The PCR products were then purified with the assistance of shrimp alkaline phosphatase (SAP) and exonuclease I. The SNaPshot PCR reaction mix comprised 5 μl of SNaPshot Multiplex Kit, 2 μl of ultrapure H_2_O, 2 μl of the purified PCR products and 1 μl of the primer mix. Extension PCR was performed as follows: 1 cycle of 96°C/1 min.; 28 cycles of 96°C/10 sec., 55°C/5 sec. and 60°C/30 sec.; and 1 cycle of 4°C/2 min. After further purification by SAP, the final products were analysed with an ABI 3730xl DNA Analyzer and GeneMapper 4.1 (Applied Biosystems, Carlsbad, CA, USA).

### Dual‐luciferase reporter assay

According to the *Homo sapiens* chromosome 17, GRCh38.p7 primary assembly, a 2‐kb sequence upstream of the SMG6 transcription start site containing the T or G alleles at rs4523957, was cloned and individually ligated into pGL3 basic to create the plasmids pSMG6‐Promoter‐Wildtype and pSMG6‐Promoter‐Mutant, and both of these plasmids were amplified in DH5α cells. Their positive clones were confirmed by sequencing. HEK‐293T cells were plated at 2 × 10^4^ cells per well in 24‐well dishes, and 24 hrs later, the cells were cotransfected with X‐tremeGENE HP reagent (Roche, Basel, Switzerland). Each cotransfection reaction was replicated four times and contained 1 μg of pGL3 basic as the negative control (NC), 1 μg of pGL3 promoter as the positive control (PC), 1 μg of pSMG6‐Promoter‐Wildtype (wild‐type), 1 μg of pSMG6‐Promoter‐Mutant (Mutant) and 50 ng of pRL plasmid, which served as an internal reference. After 24 hrs, the cotransfected cells were processed using a Dual‐Luciferase^®^ Reporter Assay System (Promega, Madison, WI, USA), and the firefly luciferase activity was measured using a microplate luminometer (BioTek, VT, USA) and normalized to the Renilla luciferase activity. The mutant impact of rs4523957 T > G was calculated by dividing the averaged firefly/Renilla ratio of the mutant construct by the averaged firefly/Renilla ratio of the wild‐type construct.

### Epilepsy rat model

A total of 15 male Sprague Dawley (SD) rats were obtained from the Animal Center of Guangdong Medical University, Zhanjiang, China. All rats were bred at a temperature of 22–26°C and humidity of 55–65%. The light–dark cycle was consistent with a natural day‐and‐night cycle. After a 1‐week adaptation to the experimental environment with free access to food and water, the SD rats (310 ± 32 g) were used in the following experiments: 10 rats were randomly selected to be treated with pentetrazole (PTZ, 60 mg/kg body weight, i.p.; Sigma‐Aldrich, St. Louis, MO, USA). According to the Racine scale 23, 24, the severity of epileptic seizures was classified into the five following levels: (*i*) Twitching of facial muscle; (*ii*) Nodding of the head; (*iii*) Unilateral forelimb with lifting or clonus; (*iv*) Bilateral forelimb with clonus when standing; (*v*) Falling when standing or twisting. During an observation time of 2 hrs, PTZ was administered every 20 min. (10 mg/kg body weight, i.p.) until seizures up to level 4 were induced or the total dose of PTZ reached 90 mg/kg body weight. Finally, nine rats exhibited seizures up to level 4 that lasted 60 min., and these were immediately enrolled in the experimental group and administered an injection of diazepam (10 mg/kg body weight, i.p.; Sigma‐Aldrich) every 5 min. until seizure cessation to reduce unexpected deaths before killed. The remaining fine rats were classified as the control group. All animal experiments performed in this study were conducted according to the Guide for the Care and Use of Laboratory Animals (Ministry of Science and Technology of China, 2006) and approved by the Animal Ethics Committee of Guangdong Medical University, Zhanjiang, China.

### Molecular experiments

After a 2‐hrs observation period, all of the rats belonging to the experimental and control groups were skilled by decapitation under deep anaesthesia (3% chloral hydrate, 10 ml/kg body weight, i.p.; Sigma‐Aldrich). The hippocampi of nine experimental rats and four control rats were rapidly collected for the following experiments: (*i*) Real‐time quantitative PCR (qPCR): Total RNA was isolated using an RNA extraction kit (QIAGEN Sciences, Germantown, USA) and subjected to reverse transcription using a First Strand cDNA Synthesis Kit (Thermo Fisher Scientific, Waltham, USA) in accordance with the manufacturer's instructions. The cDNA products were then amplified using a Light‐Cycler 480 sequence detector system (Roche Applied Science, Penzberg, Germany), and the following specific primers were used in the real‐time quantitative PCR: SMG6 forward primer, 5′‐GCAGTGTCCTCGGTGGTAAT‐3′, and reverse primer, 5′‐GGTGTAACGCTGGGAAGGTA‐3′. The relative expression levels of SMG6 were then calculated using the 2^−ΔΔCT^ method. (*ii*) Enzyme‐linked immunosorbent assay (ELISA): The SMG6 concentration in the hippocampus was measured using an ELISA kit (R&D Systems, Minneapolis, MN, USA) according to the manufacturer's instructions. The absorbance was detected with an ELISA reader (Bio‐Rad Laboratories, Hercules, CA, USA).

In addition to qPCR and ELISA, immunohistochemistry (IHC) was performed as follows: one experimental rat and one control rat under deep anaesthesia were cardially perfused with physiological saline followed by 4% paraformaldehyde. Their brains were then collected and fixed in 4% paraformaldehyde at 4°C for 20 hrs. After dehydration and paraffin embedding, the specimens were sectioned into 4‐μm‐thick slices for histological staining. Paraffin‐embedded slices were dewaxed using xylene and ethanol. Deparaffinized slices were rinsed with water and exposed to microwave, and endogenous peroxidase was then blocked with 3% hydrogen peroxide. After three 5‐min. washes with phosphate buffer saline (PBS), the slices were blocked with 3% BSA. Subsequently, the primary antibody (SMG6 polyclonal antibody, ABclonal, Boston, MA, USA) was diluted in blocking solution and applied overnight at 4°C. After three 5‐min. Washes in PBS, the slices were incubated with the secondary antibody, washed, and developed with DAB, and washed to stop the reaction once a brown colour appeared. Finally, the slices were then counterstained with haematoxylin, dehydrated and mounted.

### Bioinformatic analyses

First, 2‐kb sequences upstream of the SMG6 transcription start site containing T or G alleles at rs4523957 were attained from the UCSC Genome Browser developed and maintained by the Genome Bioinformatics Group, and the obtained sequences were used to predict binding sites of transcriptional factors (TFs) using Alibaba 2.1, which was developed by Niels Grabe, with the aim of identifying the different TFs surrounding the polymorphic site between the SMG6 promoter with T or G alleles at rs4523957. GSE63808 was then downloaded from the GSE data sets, which were obtained using an Illumina HumanHT‐12 V3.0 Expression BeadChip array 25. GSE63808 is composed of surgically acquired hippocampi from 129 TLE patients. After a series of background adjustments and quantile normalization, GSE63808 was used for gene co‐expression analyses of the neighbouring genes SRR and SMG6 using R 3.2.5 (R Foundation for Statistical Computing, Vienna, Austria) and an R package (WGCNA, Weighted Gene Co‐Expression Network Analysis) 26.

### Statistical analyses

Variable data are displayed as the means ± standard deviation (S.D.) and were analysed with Student's *t*‐test, and attribute data were analysed using Chi‐squared test or Fisher's exact test. Logistic regression was used to correct for bias introduced by confounding factors, such as age, gender and disease duration, and *q* values were obtained to adjust false‐positive results obtained from multiple statistics *via* the Bonferroni correction. Statistical tests were mainly performed using SPSS 19.0 (IBM, New York, NY, USA), and *P* ≤ 0.05 was considered significant. In addition, power and haplotype analyses were performed with Quanto 1.2 (University of Southern California, Los Angeles, CA, USA) and Haploview 4.2 (Daly Lab, Cambridge, MA, USA), respectively.

## Results

### Basic characteristics of enrolled cohorts and genotyping data

In total, 496 TLE patients and 528 healthy individuals were recruited in this study. No significant differences in gender or age were found between the TLE cases and the healthy controls in cohorts 1, 2 and 1 + 2 (all *P* values > 0.05). The gender, age, disease duration and severity of disease for cohort 1 + 2 are displayed in Table 1.

**Table 1:** Basic characteristics of enrolled cohorts

	Cases	Controls	*P* values
Gender (male/female, *n*)			
Cohort 1	170/165	185/140	0.112
Cohort 2	79/82	104/99	0.682
Cohort 1 + 2	249/247	289/239	0.147
Age (mean±S.D., years)			
Cohort 1	31.2 ± 14.7	31.0 ± 9.7	0.862
Cohort 2	33.5 ± 14.9	32.7 ± 15.6	0.617
Cohort 1 + 2	31.5 ± 14.8	31.7 ± 12.3	0.743
Other characteristics in cohort 1 + 2			
Disease duration (mean±S.D., years)	11.2 ± 10.0	–	–
Severity of disease			
Seizure frequencies (mean±S.D., times/month)	6.7 ± 2.7	–	–
Drug response (sensitive/resistant patients, *n*)	250/246	–	–

In this study, all recruited cases and controls were successfully genotyped for three SRR SNPs (rs4523957, rs8081273 and rs7222251), but we failed to identify polymorphisms at rs408067. The frequency distributions of rs4523957, rs8081273 and rs7222251 SNPs comply with Hardy–Weinberg equilibrium in cohorts 1 and 2. Power analyses showed that this study would have 94.1%, 98.1% and 93.7% power to detect a recessive inheritance model for rs4523957, rs8081273 and rs7222251, respectively, with an odds ratio (OR) of 2.0 at a significance level of 0.05.

### Analyses of the association of the SRR SNPs between the cases and controls

In cohorts 1 and 2, the frequencies of the GG genotype at rs4523957 T > G were consistently decreased in the TLE cases compared with the healthy controls (Table 2: *P* = 0.048 and *P* = 0.034, respectively). Moreover, the same trend was observed for the heterozygous mutation after Bonferroni correction in cohort 1 + 2 (Table 3: OR = 1.891, *P* = 0.006 and *q* = 0.050), which confirms the findings in cohorts 1 and 2. These results indicate that the GG genotype could be associated with protection against TLE. However, no consistent differences were observed for the other SNPs (rs8081273 and rs7222251) in cohorts 1, 2 and 1 + 2.

**Table 2:** Inheritance models of the SRR SNPs in cohorts 1 and 2

	Cohort 1		*P* values	Cohort 2		*P* values
	Cases *n* (%)	Controls *n* (%)		Cases *n* (%)	Controls *n* (%)	
rs4523957 T > G						
TT/TG/GG	170(50.7)/141(42.1)/24(7.2)	166(51.1)/123(37.8)/36(11.1)	0.394	85(71.4)/68(26.1)/8(2.5)	102(69.0)/78(27.6)/23(3.4)	0.180
TT/TG+GG	170(50.7)/165(49.3)	166(51.1)/159(48.9)	0.981	85(71.4)/76(28.6)	102(69.0)/101(31.0)	0.608
TT+TG/GG	311(92.8)/24(7.2)	289(88.9)/36(11.1)	0.048	153(97.5)/8(2.5)	180(96.6)/23(3.4)	0.034
rs8081273 T > C						
TT/TC/CC	152(45.4)/153(45.7)/30(9.0)	134(41.2)/145(44.6)/46(14.2)	0.067	72(93.2)/74(5.6)/15(1.2)	82(83.7)/93(13.9)/28(2.5)	0.216
TT/TC+CC	152(45.4)/183(54.6)	134(41.2)/191(58.8)	0.270	72(93.2)/89(6.8)	82(83.7)/121(16.3)	0.408
TT+TC/CC	305(91.0)/30(9.0)	279(85.8)/46(14.2)	0.034	146(98.8)/15(1.2)	175(97.5)/28(2.5)	0.196
rs7222251 T > C						
TT/TC/CC	171(51.0)/134(40.0)/30(9.0)	167(51.4)/132(40.6)/26(8.0)	0.907	82(93.2)/64(5.6)/15(1.2)	105(83.7)/82(13.9)/16(2.5)	0.744
TT/TC+CC	171(51.0)/164(49.0)	167(51.4)/158(48.6)	0.982	82(93.2)/79(6.8)	105(83.7)/98(16.3)	0.876
TT+TC/CC	305(91.0)/30(9.0)	299(92.0)/26(8.0)	0.760	146(98.8)/15(1.2)	187(97.5)/16(2.5)	0.636

Remarks: The *P* values have been adjusted for gender and age.

**Table 3:** Inheritance models of the SRR SNPs in cohort 1 + 2

	Cases *n* (%)	Controls *n* (%)	ORs (95% CI)	*P* values	*q* values
rs4523957 T > G					
TT/TG/GG	255(70.2)/209(27.4)/32(2.4)	268(68.7)/201(28.0)/59(3.2)	1.147(0.949–1.387)	0.156	1.401
TT/TG+GG	255(70.2)/241(29.8)	268(68.7)/260(31.3)	0.968(0.757–1.238)	0.797	7.171
TT+TG/GG	464(97.6)/32(2.4)	469(96.8)/59(3.2)	1.891(1.203–2.972)	0.006	0.050
rs8081273 T > C					
TT/TC/CC	224(92.5)/227(6.3)/45(1.2)	216(84.9)/238(13.6)/74(1.5)	1.233(1.025–1.483)	0.026	0.235
TT/TC+CC	224(92.5)/272(7.5)	216(84.8)/312(15.2)	0.837(0.653–1.073)	0.160	1.443
TT+TC/CC	451(98.8)/45(1.2)	454(98.5)/74(1.5)	1.642(1.018–2.435)	0.014	0.122
rs7222251 T > C					
TT/TC/CC	253(92.5)/198(6.3)/45(1.2)	272(84.9)/214(13.6)/42(1.5)	0.970(0.801–1.176)	0.760	8.330
TT/TC+CC	253(92.5)/243(7.5)	272(84.8)/256(15.2)	1.012(0.790–1.297)	0.926	6.839
TT+TC/CC	451(98.8)/45(1.2)	486(98.5)/42(1.5)	0.886(0.570–1.379)	0.593	5.336

Remarks: The ORs and *P* values have been adjusted for gender and age, and the *q* values were calculated using the Bonferroni correction.

As shown in Table 4, a haplotype block (rs4523957‐rs8081273‐rs7222251), which was approximately 17 kb in length, was constructed using Haploview 4.2 based on the three intronic SNPs in the SRR gene region. The frequencies of the SRR haplotypes were then compared between the cases and controls in cohort 1 + 2. The results revealed that the case ratio of the GCT haplotype was lower than the control ratio (4.3% *versus* 6.8%, *P* = 0.003), and this finding was further validated after Bonferroni correction (*q* = 0.027), which indicates that the GCT haplotype is a protective marker against TLE.

**Table 4:** Haplotypes of the SRR SNPs in cohort 1 + 2

	Haplotypes	Frequency ratios (%)	Case ratios (%)	Control ratios (%)	*P* values	*q* values
rs4523957‐rs8081273‐rs7222251 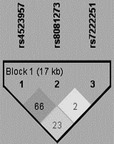	TTT	45.5	46.9	44.3	0.235	1.878
	GCT	14.2	11.8	16.3	0.003	0.027
	TTC	13.7	13.8	13.7	0.955	7.636
	TCT	10.0	9.9	10.0	0.928	7.422
	GCC	8.4	8.3	8.4	0.955	7.637
	GTC	4.7	5.0	4.3	0.460	3.682
	TCC	1.8	1.9	1.8	0.859	6.871
	GTT	1.7	2.3	1.1	0.036	0.285

Remarks: The *q* values were calculated with the Bonferroni correction.

### Genetic effects of the GG genotype at rs4523957 on disease severity

**Figure 1:**
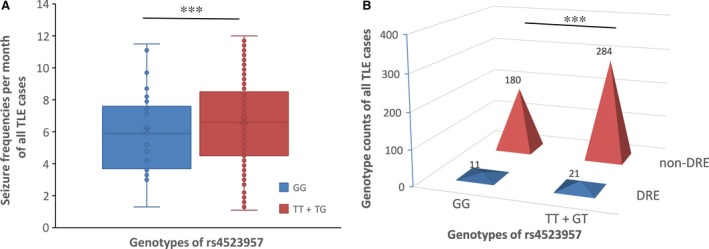
Genetic effects of the GG genotype at rs4523957 on disease severity. (**A**) The seizure frequencies for the GG genotype and the TT + TG genotypes were 6.1 ± 2.6 and 6.7 ± 2.7 per month, respectively. (**B**) The drug‐resistant incidences for the GG genotype and the TT + TG genotypes were 5.8% and 6.9%, respectively. ***P < 0.001.

Based on the findings obtained from the comparisons of the cases and controls, we further evaluated the genetic effects of the heterozygous mutation at rs4523957 T > G on disease severity in cohort 1 + 2. As shown in Figure 1, the seizure frequency per month was lower in carriers of the GG genotype compared with carriers of the TT + TG genotypes after adjustment for gender and disease duration (*P* = 0.000). The drug‐resistant incidence was reduced in carriers of the GG genotype compared with carriers of the TT + TG genotypes after adjustment for gender and disease duration (*P* = 0.000). Both of these findings support a protective role of the GG genotype in disease severity.

### Impacts of rs4523957 mutation on the neighbouring SMG6 gene

**Figure 2:**
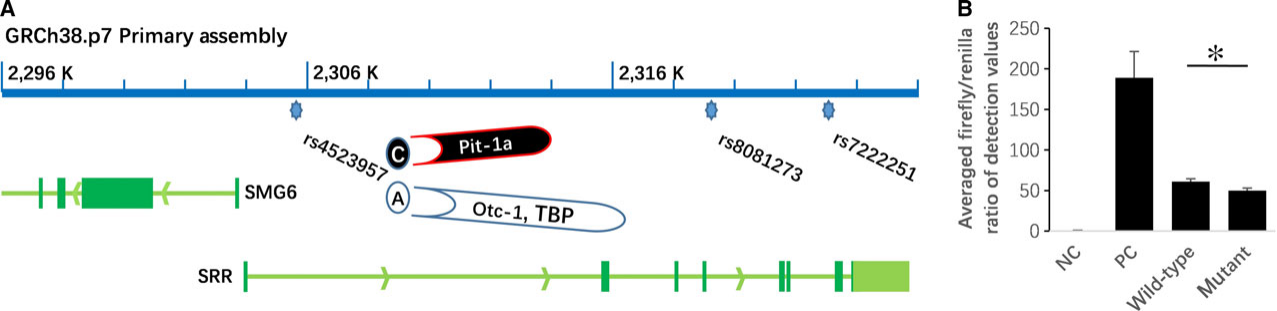
Impacts of rs4523957 mutation on the neighbouring SMG6 gene. (**A**) Unlike the other two intronic SNPs (rs8081273 and rs7222251), rs4523957 is located in the promoter region of SMG6 and in the first intron of SRR. Moreover, different profiles of TFs for the mutant C allele (Pit‐1a) and the wild‐type A allele (Otc‐1 and TBP) at rs4523957 (chromosome 17, GRCh38.p7 (2305605, complement)) were predicted by Alibaba 2.1, indicating that rs4523957 is a functional SNPs for the SMG6 gene (chromosome 17, GRCh38.p7 (2059839.2303836, complement). (**B**) Based on the averaged firefly/Renilla ratios obtained for the pGL3 basic negative control (NC) and the pGL3 promoter positive control (PC), the ratios obtained for pSMG6‐Promoter‐Wildtype (2‐kb sequence upstream of the transcription start site; chromosome 17, GRCh38.p7 (2061839..2359840, complement), A allele at rs4523957, wild‐type) and pSMG6‐Promoter‐Mutant (2‐kb sequence upstream of the transcription start site; chromosome 17, GRCh38.p7 (2061839..2359840, complement), C allele at rs4523957, mutant) constructed for rs4523957 are 61 ± 4 and 50 ± 3, respectively. *P < 0.05.

As shown in Figure 2, rs4523957 is located in the promoter region of SMG6, as well as in the first intron of the SRR gene. To identify the impact of rs4523957 mutation on the SMG6 gene promoter, we synthesized reporter gene constructs containing A (wild‐type) or C (mutant) alleles at rs4523957 (chromosome 17, GRCh38.p7 (2305605, complement)) in the context of the full‐length promoter of SMG6 (2‐kb sequence upstream of the transcription start site; chromosome 17, GRCh38.p7 (2061839..2359840, complement)). A dual‐luciferase assay revealed an approximate 22% decrease in the activity of the mutant construct compared with that of the wild‐type construct, which strongly indicates that variation at rs4523957 could down‐regulate SMG6 transcription.

### Expression patterns of SMG6 and its potential link with SRR in TLE

**Figure 3:**
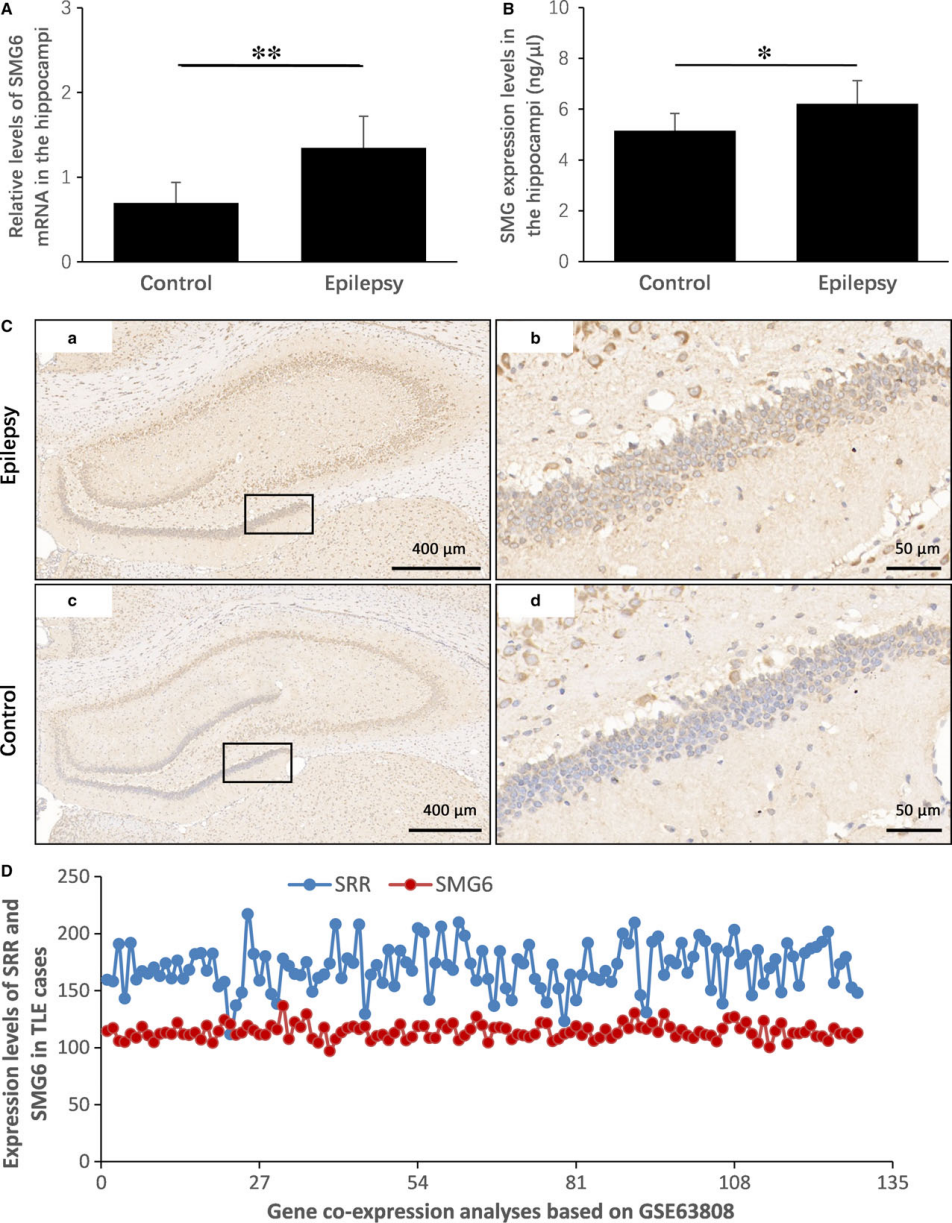
Expression patterns of SMG6 and its potential link with SRR in TLE. (**A**) As determined through qPCR, the relative levels of SMG6 mRNA in the hippocampi of the control and epilepsy groups were 0.7 ± 0.2 and 1.3 ± 0.4, respectively. (**B**) As determined through ELISA, the concentrations of SMG6 protein in the hippocampi of the control and epilepsy groups were 5.2 ± 0.7 and 6.2 ± 0.9 ng/μl, respectively. (**C**) IHC images of global and local regions of the hippocampi. Haematoxylin staining was used to mark the cell nucleus (blue), and tissues with immunostaining showed SMG6 expression (brown). *P < 0.05, **P < 0.01. (**D**) Gene co‐expression analyses of SRR and SMG6 in the surgically acquired hippocampi of 129 patients with TLE. GSE63808 was used in the gene co‐expression analyses, and the correlation coefficient of SRR and SMG6 was 0.022.

To determine whether SMG6 is involved in epileptic activities, we established a rat epilepsy model based on a protocol involving PTZ induction. The expression of SMG6 mRNA and protein in the hippocampi was detected through qPCR and ELISA, respectively. Compared with control rats, the epilepsy model exhibited an approximate 52% increase (*P* = 0.006) in SMG6 mRNA expression (Fig. 3A), which is similar to the increase found for SMG6 protein (approximately 14%, *P* = 0.010; Fig. 3B). In addition, IHC confirmed the abnormal expression of SMG6 in the epilepsy model (Fig. 3C), indicating that hyperfunction of SMG6 is involved in epileptic seizures.

In eukaryotic genomes, genes with related biological activities tend to cluster and function in a cooperative manner 27, 28. To determine whether an interactive link between the neighbouring genes SRR and SMG6 exists in TLE, TLE array data (GSE63808) were downloaded from the online GSE data sets and used for gene co‐expression analyses in this study. The correlation coefficient between SRR and SMG6 was extremely low (Fig. 3D), indicating no biological link between SRR and SMG6 in TLE.

## Discussion

To the best of our knowledge, TLE is the most common partial epilepsy and the primary origin of intractable seizures, but the currently available drugs are not effective for all patients, likely because of the high genetic heterogeneity of TLE as well as untraceable environmental effects. Some genetic markers have been identified 29, 30, but these only account for less than ten per cent of patients with TLE, which indicates that the heterogeneity of TLE needs to be further explored in future studies. In the present study, several SNPs of the SRR gene were specifically selected with the aim of gaining some insights regarding the genetic regulation of SRR in TLE. Here, we first observed that the GG genotype at rs4523957 and the GCT haplotype (rs4523957‐rs8081273‐rs7222251) were protective markers against TLE. Moreover, carriers of the GG genotype presented decreased disease severity than carriers of the TT+TG genotypes at rs4523957, as determined through analyses of seizure frequencies and drug response in all TLE cases in cohort 1 + 2.

Notably, the protective effect of variation at rs4523957 is difficult to explain because the rs4523957 locus is in the first intron of SRR and thus often classified as a non‐functional polymorphic zone for protein‐coding genes. Intriguingly, rs4523957 is also located in the promoter region of the SMG6, a neighbouring gene of SRR, which prompted us to question whether the mutant effects at rs4523957 are mediated by SMG6. Subsequently, we attempted to predict TFs and observed different profiles for the wild‐type (Otc‐1 and TBP) and mutant (Pit‐1a) alleles at rs4523957. Among these TFs, TBP functions at the core of the DNA‐binding multiprotein factor TFIID and participates in the activation of genes transcribed by RNA polymerase II, indicating a potential genetic modulation of SMG6 expression. More importantly, compared with the wild‐type construct, the mutant construct exhibited significant down‐regulation in activity, as demonstrated through a dual‐luciferase assay. In addition, multiple evidence from qPCR, ELISA and IHC analyses showed that the expression of SMG6 was consistently increased in an epilepsy rat model. These findings provide the first evidence of SMG6 hyperfunction in TLE.

NMD is a mRNA surveillance system and is responsible for limiting the synthesis of potentially harmful truncated proteins 23. An estimated 30% of known human disease‐associated mutations originate from premature translation termination codon (PTC)‐containing mRNAs 24, and several PTC mutations, such as S326fs328X in the gamma‐amino butyric acid type A receptor alpha 1 subunit, have been observed to be associated with epileptic seizures 19, 20. SMG6 plays a crucial role in NMD initiation by providing endonuclease activity near PTC. According to dbSNP, SRR, a neighbouring gene of SMG6, contains approximately 1289 SNPs in its gene region, and only mutations at rs780526426 and rs138106032 result in PTC. However, PTC‐related mutations are usually rare in human beings and are therefore an unlikely dominant mechanism for the protective effect of variation at rs4523957 against TLE observed in the study. Moreover, our gene co‐expression analyses revealed that the correlation coefficient of SRR and SMG6 is extremely low, indicating that SRR and SMG6 function independently in TLE. A recent study suggested that SMG6 goes far beyond its classic role and functions in global gene expression by regulating error‐free transcripts 31, but its implications in epileptic activities remain to be clarified.

In addition to NMD, the telomere is crucial for biological activities. In general, senescence is activated as soon as the telomere shortens to a critical extent during cell division; thus, telomere shortening is considered a determinant of neurodegenerative diseases. Moreover, telomere shortening could even be accelerated by inflammation responses, and this effect likely occurs because of excessive differentiation of neuroprogenitor cells into inflammatory cells, such as microglia and astrocytes. Interestingly, epileptic seizures are a common complication in ageing diseases, such as Alzheimer's disease 32, and inflammation also acts as a pathological sign of TLE 33. These observations indicate the potential role of telomere shortening in TLE. SMG6 is an element of the telomerase ribonucleoprotein complex responsible for telomere replication, but the association between SMG6 and epileptic seizures has not yet been determined.

Notably, telomerase is a key molecule responsible for adding telomere repeats to chromosome ends. Under normal conditions, the activity of telomerase is high in brain tissues during embryonic development and is almost undetectable in adulthood. Intriguingly, telomerase expression has been found to be up‐regulated in the hippocampus of mice with kainite‐induced epilepsy and is similar to the time course of microglial activation 34. Significant telomerase activity was also observed in neuroprogenitor cells derived from the hippocampi of patients with TLE 35. More importantly, telomerase transgenic mice showed a lower threshold for electrically induced seizures, which could be reversed by the NMDA receptor antagonist memantine 36. These lines of evidence indicate a new role for telomerase in TLE that involves regulating NMDA receptor‐mediated excitatory disorders. Because SMG6 is a cofactor of telomerase in the maintenance of telomere length, the possibility that SMG6 functions together with telomerase in TLE by regulating the activity of NMDA receptors should be investigated in future studies.

The following limitations of the study should be mentioned: The variation at rs408067 is potentially functional because its locus is found in the SRR promoter region, but this SNP could not be genotyped because of the presence of a highly repeated CG sequence surrounding the polymorphic site, which strongly limited its combination with the extension primer. Hence, this study failed to answer the question of whether SRR SNPs are functionally involved in TLE. Interestingly, when analysing why rs408067 failed to be genotyped, we noticed the existence of a CpG island in the SRR gene promoter; thus, the methylation level and its impact on TLE should be taken into consideration in future studies. According to the dual‐luciferase assay for rs4523957, the activity of the mutant construct showed an approximate 22% decrease compared with that of the wild‐type construct, but SMG6 expression in the pathological foci of carriers of the mutation could not be validated because corresponding brain specimens are not available. Moreover, the prediction of TFs is not consistent among Alibaba 2.1, JASPAR and ALGGEN; thus, we did not identify the role of TBP, Pit‐1a and otc‐1, which were predicted by Alibaba 2.1, in the impact of rs4523957 mutation on the SMG6 promoter. In our opinion, further systematic experiments, such as chromatin immunoprecipitation, mass spectrometry and protein analysis, should be performed to identify transcription factors that have different functions in individuals with wild‐type and mutant alleles at rs4523957 (chromosome 17, GRCh38.p7 (2305605, complement)) in the SMG6 promoter. In addition, the different human races contribute to a substantial amount of genetic heterogeneity, that is, the minor allele frequencies of rs4523957 are different in European and Asian (38.5% *versus* 23.2%, respectively); thus, our results, which are based on the Chinese Han population, should be generalized to other racial individuals with caution, even though the findings were validated in an independent cohort consisting of individuals from different geographic regions of China and false‐positive adjustments were made using the Bonferroni correction.

## Conclusions

In conclusion, this study provides the first demonstration that the GG genotype at rs4523957 and the GCT haplotype (rs4523957‐rs8081273‐rs7222251) are protective genetic markers against TLE. In particular, variation at rs4523957 T > G likely inhibits SMG6 transcription and plays a key role in the susceptibility to and severity of TLE. The significance of SMG6 hyperfunction in epileptic seizures should be investigated in future in‐depth studies.

## Conflict of interest

The authors declare that they have no conflict of interest.
